# A Woman’s Infertility Journey Complicated by Severe Ovarian Hyperstimulation Syndrome – A Case Report

**DOI:** 10.5070/M5.52309

**Published:** 2026-04-30

**Authors:** Sukaynah Khetani, Justin Hutchison, C Michael Lee

**Affiliations:** * Kaiser Permanente Central Valley, Department of Emergency Medicine, Modesto, CA; ^ Kaiser Permanente Central Valley, Department of Obstetrics and Gynecology, Modesto, CA

## Abstract

Ovarian hyperstimulation syndrome (OHSS) is a potentially life-threatening complication of assisted reproductive technology (ART). Here, we present the case report of a 30-year-old female undergoing infertility treatment who presented to the emergency department (ED) with nausea and vomiting, abdominal distention, and shortness of breath. On physical exam, she had notable ascites. Computed tomography (CT) of the abdomen and pelvis and pelvic ultrasound (US) revealed significant ascites and enlarged ovaries with multiple cysts. She was diagnosed with severe OHSS and admitted to obstetrics and gynecology (OBGYN) service for five days where she underwent intravenous (IV) hydration and paracentesis. This case report reviews the clinical presentation, categorization, management, and prevention of OHSS and provides examples of imaging findings consistent with the condition.

**Topics:**

Infertility, obstetrics, assisted reproductive technology, ovarian hyperstimulation, third-spacing.

**Figure:**
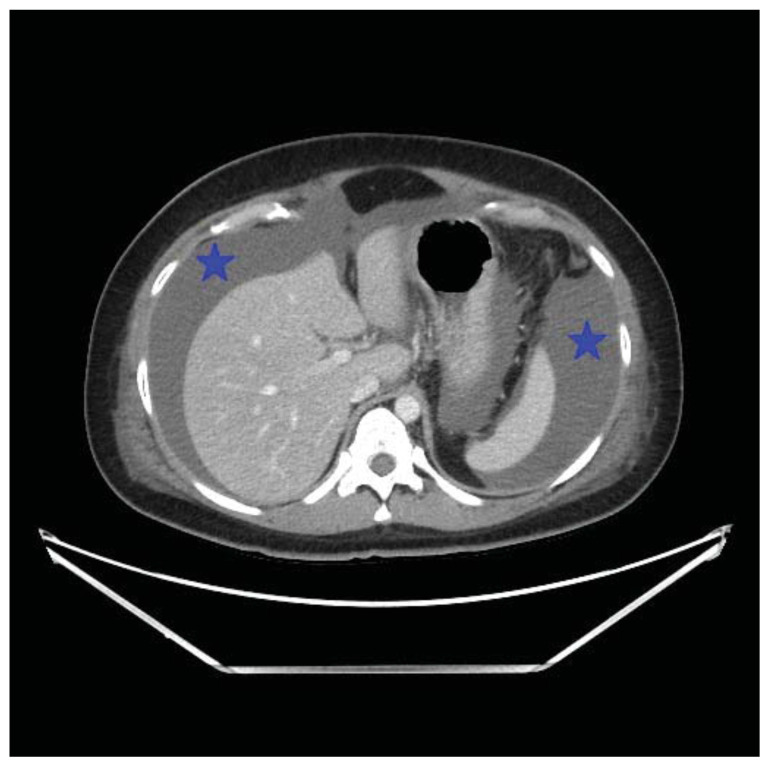


**Figure:**
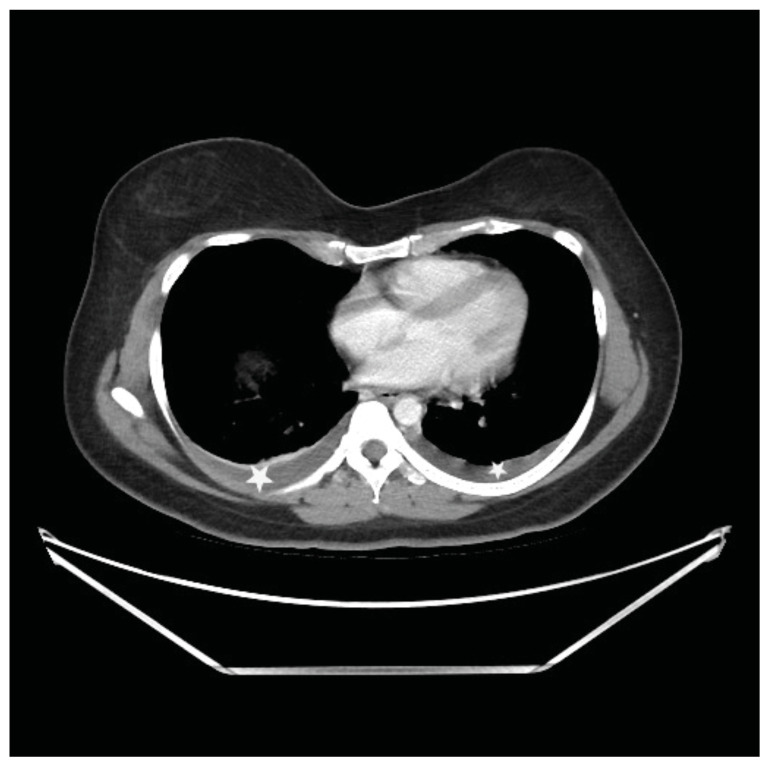


**Figure:**
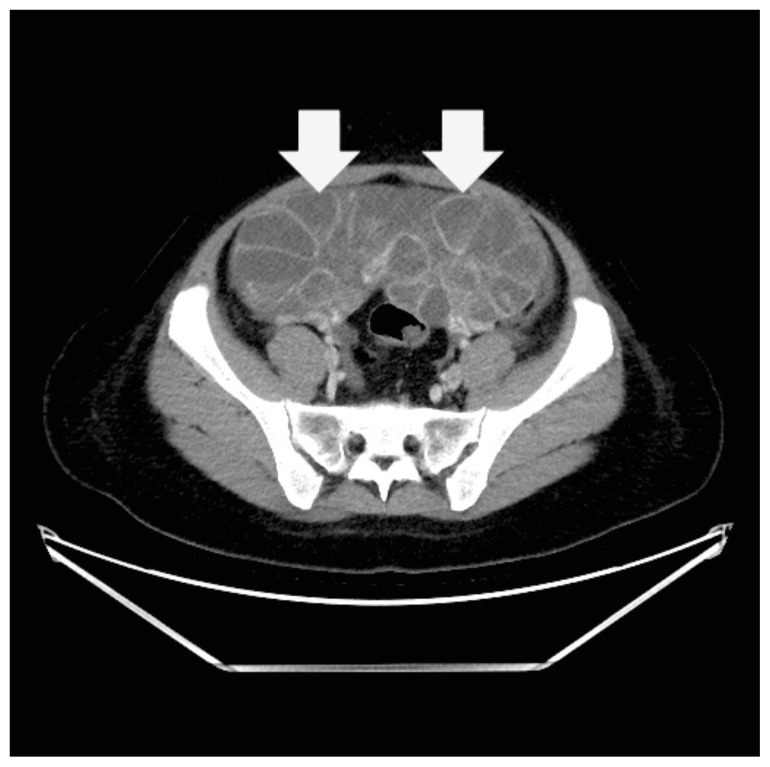


**Figure:**
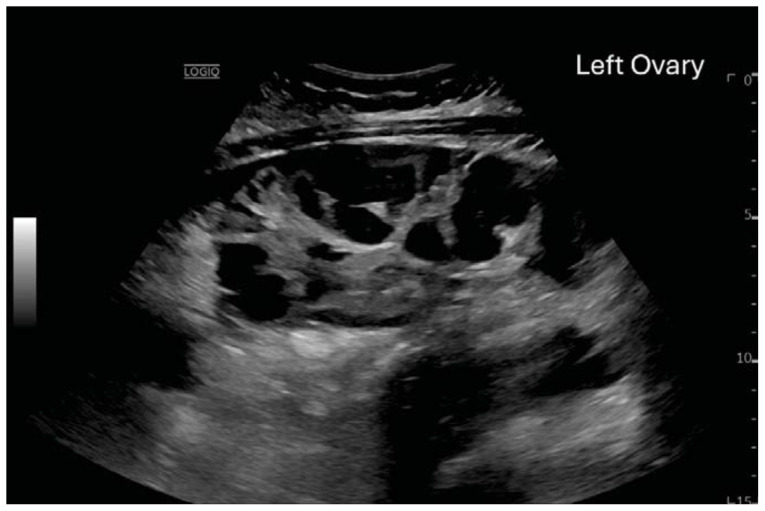


**Figure:**
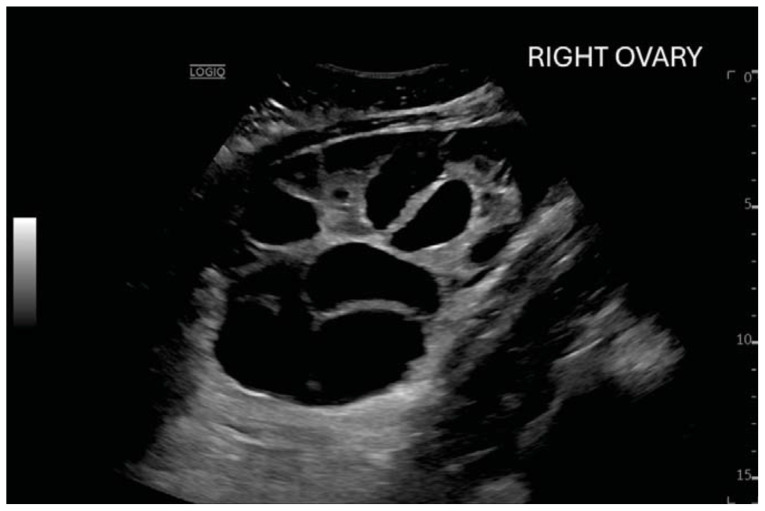


**Figure:**
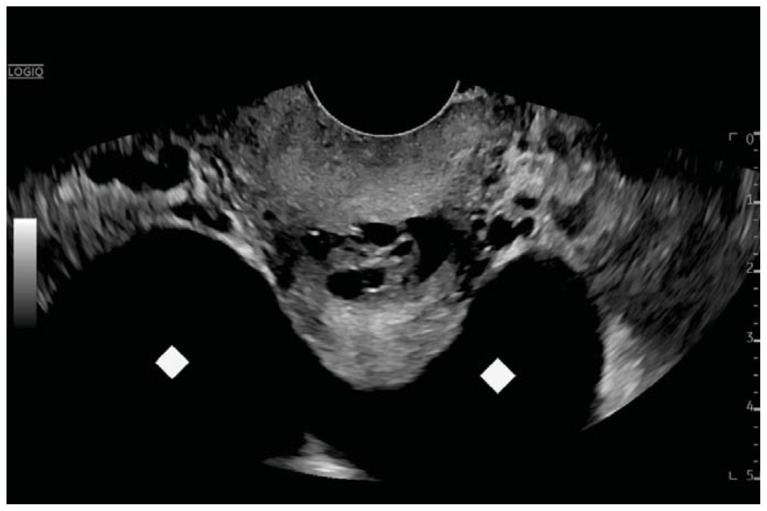


## Brief introduction

First described in 1943, ovarian hyperstimulation syndrome (OHSS) is a condition that encompasses a broad range of clinical severity (from mild to critical) with the earliest fatal cases reported in 1951.^1^,^2^,^3^ It can occur spontaneously, typically in patients with multiple gestation, hypothyroidism, and polycystic ovarian disease (PCOS), or with pituitary adenomas, follicle stimulating hormone (FSH)-secreting tumors, human chorionic gonadotropin (hCG)-secreting tumors, and other trophoblastic neoplasms.^4^–^6^ However, it is most commonly encountered as an iatrogenic complication of ART which usually includes some form of controlled ovarian stimulation.^4^,^6^

The syndrome is characterized by ovarian enlargement with multiple cysts, ascites, and extravasation of intravascular fluid into the third space secondary to increased capillary permeability.^1^,^2^ Symptoms of OHSS may include abdominal pain or distention, nausea, vomiting, decreased urine output, shortness of breath, and weight gain.^5^ Because there is no consensus definition of OHSS or its severity, the exact number of cases is hard to pinpoint.^6^ It is estimated that approximately 30% of women undergoing ART will experience OHSS, but the prevalence of moderate cases is only about 3–10% while the prevalence of severe cases and critical cases are only 0.1–3% and less than 0.1% respectively.^6^ This case report highlights a severe presentation of OHSS in a 30-year-old female undergoing ART.

## Presenting concerns and clinical findings

A 30-year-old female with a past medical history of infertility secondary to polycystic ovarian syndrome (PCOS) and a partner with male infertility factor presented to the ED with shortness of breath, nausea, vomiting, abdominal pain, abdominal distention, and a weight gain of nine pounds over the past week.

Seven days prior, she had undergone ovarian stimulation with follicle-stimulating hormone (FSH) injections and oocyte retrieval yielding 43 oocytes. She began developing symptoms shortly after the procedure and was evaluated by her fertility specialist who prescribed her furosemide. Despite using furosemide for two days, her symptoms worsened prompting her to seek care in the ED.

On evaluation in the ED, the patient was afebrile with a blood pressure of 116/69 mmHg, heart rate of 138 beats per minute, a respiratory rate of 23 breaths per minute, and an oxygen saturation of 97% on room air. She appeared very uncomfortable and had difficulty breathing when asked to lie flat. Her abdomen was diffusely tender and distended with a positive fluid wave. She had no lower extremity edema.

## Significant findings

Her blood work was notable for multiple electrolyte abnormalities including a sodium of 132 mEq/L and potassium of 5.5 mEq/L. She had a leukocytosis of 19,700/mm3, an elevated hematocrit (Hct) of 50.3%, and an elevated creatinine of 1.53 mg/dL.

Computed tomography (CT) of the abdomen and pelvis demonstrated pleural effusions in the lung bases (blue stars), ascites (blue stars), and enlarged ovaries with multiple cysts/follicles (white arrows). A formal pelvic US also demonstrated large volume ascites (white diamonds) and bilateral ovarian enlargement with numerous cysts. A CT angiogram (CTA) of the chest demonstrated small bilateral pleural effusions and no obvious pulmonary embolism.

## Patient course

Her overall presentation was concerning for severe OHSS. Obstetrics-gynecology (OBGYN) was consulted and admitted the patient for close monitoring, intravenous fluid hydration, renal function monitoring, and correction of electrolytes. She was started on maintenance fluids, given venous thromboembolism (VTE) prophylaxis with low molecular weight heparin, and interventional radiology (IR) was also consulted to perform a paracentesis. 2550 milliliters of fluid were removed, and fluid analysis showed no evidence of spontaneous bacterial peritonitis.

Reproductive endocrinology and infertility (REI) specialists were also consulted and recommended a five-day course of cabergoline 0.5 milligrams (mg) vaginally and ganirelix 250 micrograms (mcg) subcutaneously daily for five days if it was available. The ganirelix was not available so the patient was only treated with cabergoline. Her hospital stay was briefly complicated when her potassium increased to 6.2 mEq/L on the second day of admission, but she was treated with furosemide, calcium gluconate, insulin, sodium bicarbonate, albuterol, and sodium zirconium cyclosilicate with resolution of hyperkalemia.

Over the course of her stay, she reported symptomatic improvement, her electrolytes and renal function normalized, and her leukocytosis resolved. No further paracentesis was required. She was subsequently discharged home to follow up with her OBGYN.

## Discussion

Infertility affects approximately 12% of women in the United States, and the number of ART cycles is increasing as patients seek assistance in becoming pregnant.^6^ It is important for emergency physicians to recognize this condition to initiate appropriate treatment.

While ART is generally considered safe, patients are at risk of developing OHSS which can precipitate significant morbidity and mortality. During ART cycles, a gonadotropin-releasing hormone (GnRH) agonist or antagonist is administered to prevent premature ovulation, and then FSH or human menopausal gonadotropin (hMG) is injected to stimulate the ovaries to produce multiple follicles.^6^,^7^ After the desired follicle count or size is reached, a trigger medication, such as hCG, GnRH agonists, or GnRH antagonists, is injected to induce final oocyte maturation.^6^,^7^

Although there is expected to be some degree of ovarian hyperstimulation in response to ART, OHSS results from an exaggerated response and is characterized by ovarian enlargement and increased vascular permeability that leads to a fluid shift from the intravascular space to the third space.^1^,^2^,^8^ The exact pathophysiology behind this third-spacing is not completely understood, but it appears to involve a cascade of cytokines and angiogenic factors being released in response to fertility hormones.^1^–^3^,^6^

A key hormone in OHSS is hCG as OHSS is rare without hCG administration.^1^,^8^ The development of OHSS after the hCG trigger is believed to be mediated primarily by vascular endothelial growth factor (VEGF) which increases endothelial cell proliferation, angiogenesis, and vascular permeability.^1^,^3^ ^9^,^10^ VEGF production has been found to increase as a response to hCG administration.^11^,^12^ Furthermore, VEGF levels are higher in women that develop OHSS.^13^ The severity of OHSS has also been linked to VEGF levels.^14^ In addition to VEGF, other factors that have been implicated include estradiol, insulin-like growth factor, transforming growth factors, epidermal growth factor, platelet-derived growth factor, interleukins, and the renin-angiotensin system (RAS).^1^,^2^,^8 ^,^11^,^15^

There have been many efforts to categorize OHSS including at least five classifications by timing of symptom onset and seven classifications by severity.^8^ The American Society for Reproductive Medicine (ASRM) defines early-onset OHSS as symptoms onset four to seven days after the injection of hCG trigger while late-onset OHSS is defined as symptoms starting at least nine days after the hCG trigger.^16^

Mild cases of OHSS typically present with abdominal distention or discomfort, mild nausea and vomiting, ovarian enlargement, and no significant laboratory features, while moderate cases include the features of mild cases plus ascites seen on imaging and may have a hematocrit (Hct) > 41% and a white blood cell (WBC) count of > 15,000/microL.^16^ Severe cases are generally characterized by clinical evidence of ascites, hydrothorax, oliguria/anuria, intractable nausea/vomiting with labs demonstrating WBC count of > 25,000/mm3, sodium < 135 mEq/L, potassium > 5 mEq/L, elevated liver enzymes, Cr > 1.6 mg/dL, and Hct > 45%.^16^ Patients with critical OHSS may have hypotension, pleural effusions, rapid weight gain, syncope, venous thrombosis, acute renal failure, arrhythmia, sepsis, and acute respiratory distress syndrome (ARDS).^16^ Imaging may be of assistance in classifying severity according to the Golan classification with severe OHSS demonstrating ovaries > 12 cm, pleural effusions, and ascites.^17^

Risk factors associated with the development of OHSS include young age (< 35 years), low body mass index, PCOS, pregnancy, previous OHSS, increased number of follicles, the retrieval of > 14 oocytes, elevated serum estradiol, and higher or repeated doses of hCG trigger.^16^

Treatment of OHSS is dependent on severity but primarily supportive because the condition is usually self-limiting and tends to resolve as serum β-hCG levels decrease.^6^ Mild and moderate cases may be treated as outpatients with a focus on symptomatic relief, adequate fluid intake, monitoring of intake and output, body weight, abdominal distention, and close follow-up.^6^,^15^ Patients should be advised to avoid non-steroid anti-inflammatory medications because they may affect renal function.^15^ Strenuous activities should be avoided but strict bedrest is unnecessary and may actually increase chance of thromboembolism.^3^,^15^

In cases of severe OHSS, admission may be indicated for severe abdominal pain, inability to tolerate oral intake, significant ascites, oliguria or anuria, hypotension, dyspnea, abnormal liver function tests, or electrolyte abnormalities, namely hyponatremia and hyperkalemia.^2^ Management in this scenario should focus on fluid balance, circulatory volume correction, and correction of electrolyte abnormalities.^2^,^6^,^15^ Normal saline or a balanced crystalloid are typically sufficient although albumin may be necessary in some instances.^6^,^15^ Hyperkalemia can be treated in a typical fashion with insulin/glucose, sodium bicarbonate, albuterol, and a potassium binder, although the use of diuretics should be approached cautiously.^3^ Diuretics may actually be harmful because many of these patients are already volume depleted intravascularly, and diuretics may also increase the risk of thrombosis.^2^,^3^,^15^ Diuresis may be beneficial in treatment of pulmonary or edema or in reducing third-spacing if used in conjunction with colloid fluids on a hemodynamically stable patient whose hemoconcentration has improved (Hct< 38%).^2^,^15^ In moderate to severe cases, anticoagulant therapy should also be considered because venous thrombosis is a potential life-threatening complication of OHSS, and low-molecular weight heparin, such as enoxaparin or dalteparin, can reduce the risk of thrombosis.^2^,^15^

Paracentesis does not necessarily have to be performed in every patient but should be considered for dyspnea, abdominal distention, and oliguria.^15^ Intensive care unit admission may be required in cases of thromboembolic complications, renal failure, or ARDS.^3^ Regardless of severity, it would be prudent to consult OBGYN and REI specialists.

Prevention of OHSS is generally preferred over treatment although there is no single method to completely prevent OHSS. 2,15 However, strategies should focus on modifying controlled ovarian stimulation protocols based on each individual’s risk factors.^9^ Given how important the hCG trigger is to OHSS development, some prevention strategies include decreasing the dose of the hCG trigger or cancelling the ART cycle by withholding hCG.^3^ Using a GnRH agonist trigger as opposed to a hCG trigger has also been shown to be an effective strategy in lowering the risk of OHSS, although it carries a risk of decreasing live birth rates.^9^ “Coasting,” which is delaying hCG administration until estradiol levels decrease to a predetermined appropriate level, is another proposed strategy, although it is unclear whether or not it is truly effective at preventing OHSS.^2^,^8^,^9^ Administering a GnRH antagonist, such as ganirelix, after hCG trigger has also been a proposed preventative measure because it decreases VEGF secretion and the severity of symptoms of OHSS.^16^ Dopamine agonists, such as cabergoline, have been shown to reduce the incidence of moderate OHSS; however, even with cabergoline, OHSS incidence is as high as 10.8%, and it has not been shown to decrease incidence of severe OHSS.^2^,^8^ Other medications that have been shown to potentially reduce the risk of OHSS include metformin, aspirin, calcium, albumin, and hydroxyethyl starch solution.^2^,^9^,^10^ However, there is no definitive consensus because the ASRM notes that some of these strategies are strongly recommended with level A strength of evidence while others have weak recommendations with level C strength of evidence.^16^

The patient in this case presentation had multiple findings consistent with severe OHSS based on ASRM guidelines. She had intractable nausea and vomiting, severe abdominal pain, pleural effusions, clinical evidence of ascites, potassium > 5 mEq/L, and a Hct >45%. Overall, the treatment that she received correlates well with recommendations in the literature since the in-patient team focused on her fluid status and correcting electrolyte abnormalities. She additionally underwent paracentesis and received VTE prophylaxis. Although dopamine agonists and GnRH antagonists can be used to decrease risk of OHSS, it is unknown whether she had been on these medications prior to developing OHSS because she received fertility treatment at an outside facility although she received these medications during admission. Ultimately, the patient’s condition improved, and she was discharged home. Upon further chart review, she did not have any recurrence of OHSS.

In summary, OHSS is a potentially serious complication of ART that can have life-threatening consequences. Although much research is still required to better understand, manage, and prevent OHSS, it is important for emergency physicians to quickly identify it and request consultation in managing this complex condition.

## Supplementary Information
























