# Supplementary figures and images for: A Woman’s Infertility Journey Complicated by Severe Ovarian Hyperstimulation Syndrome – A Case Report
Source: J Educ Teach Emerg Med. 2026 Apr 30;11(2):V23–9. doi: 10.5070/M5.52309 (PMC13152376; doi:10.5070/M5.52309)

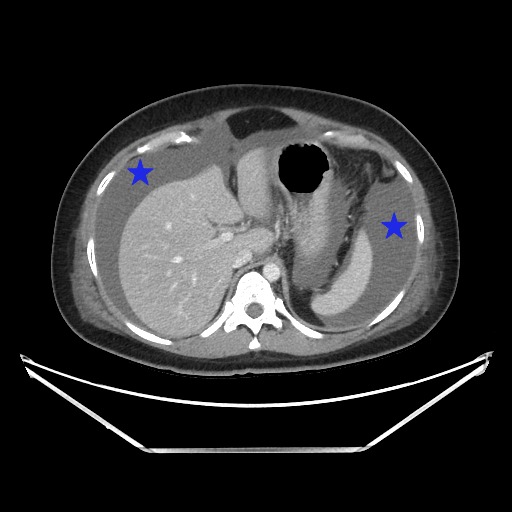

Supplement: Supplementary file 1 [file 11-2-V23-Supp1.JPG]

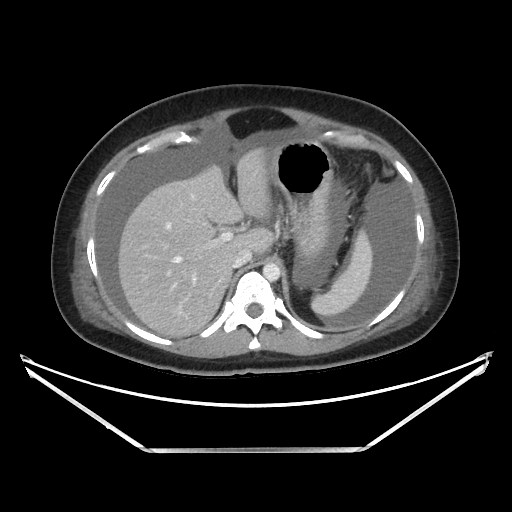

Supplement: Supplementary file 2 [file 11-2-V23-Supp2.jpg]

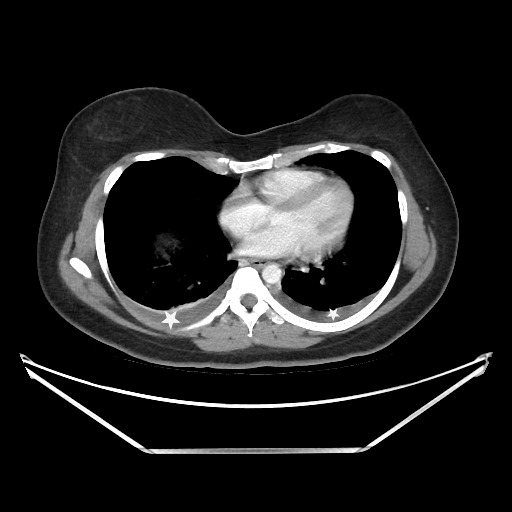

Supplement: Supplementary file 3 [file 11-2-V23-Supp3.JPG]

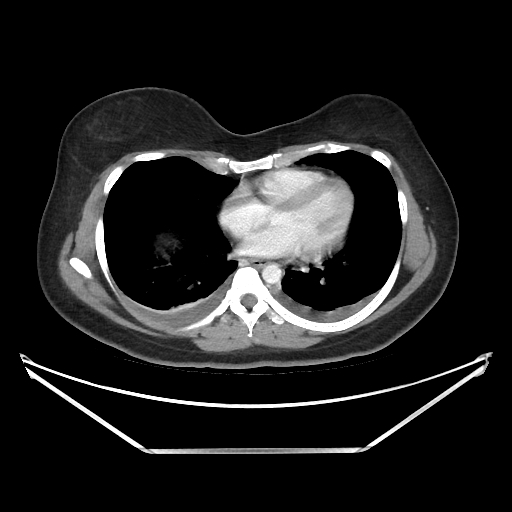

Supplement: Supplementary file 4 [file 11-2-V23-Supp4.jpg]

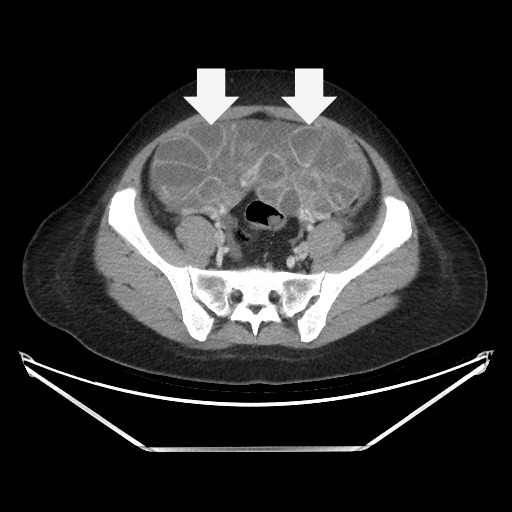

Supplement: Supplementary file 5 [file 11-2-V23-Supp5.JPG]

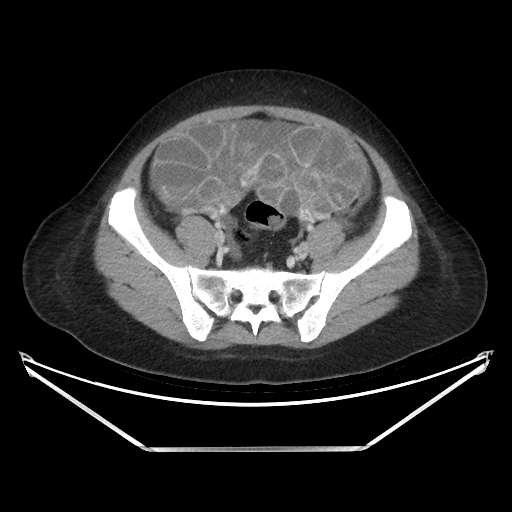

Supplement: Supplementary file 6 [file 11-2-V23-Supp6.jpg]

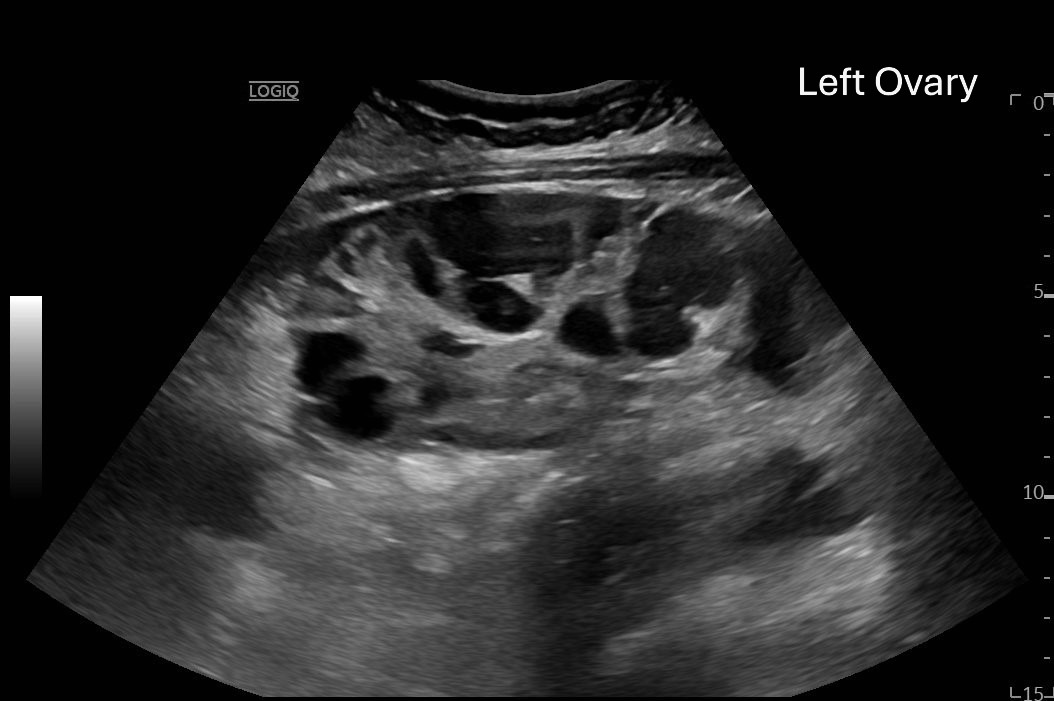

Supplement: Supplementary file 7 [file 11-2-V23-Supp7.JPG]

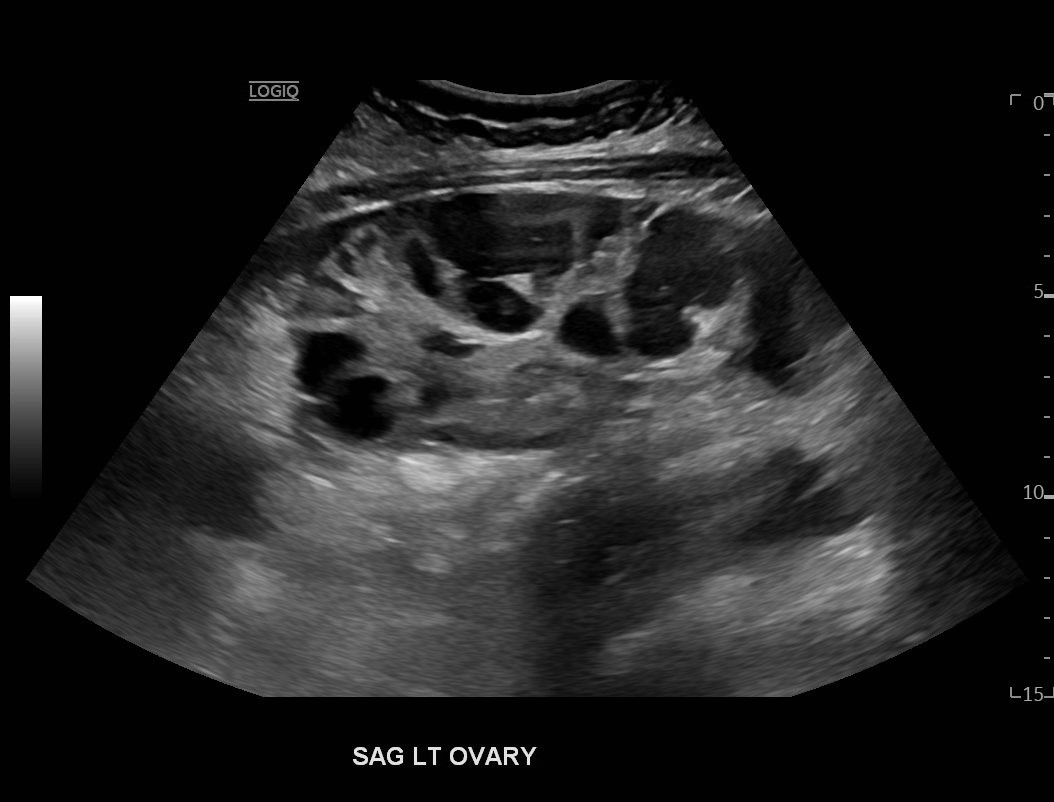

Supplement: Supplementary file 8 [file 11-2-V23-Supp8.jpg]

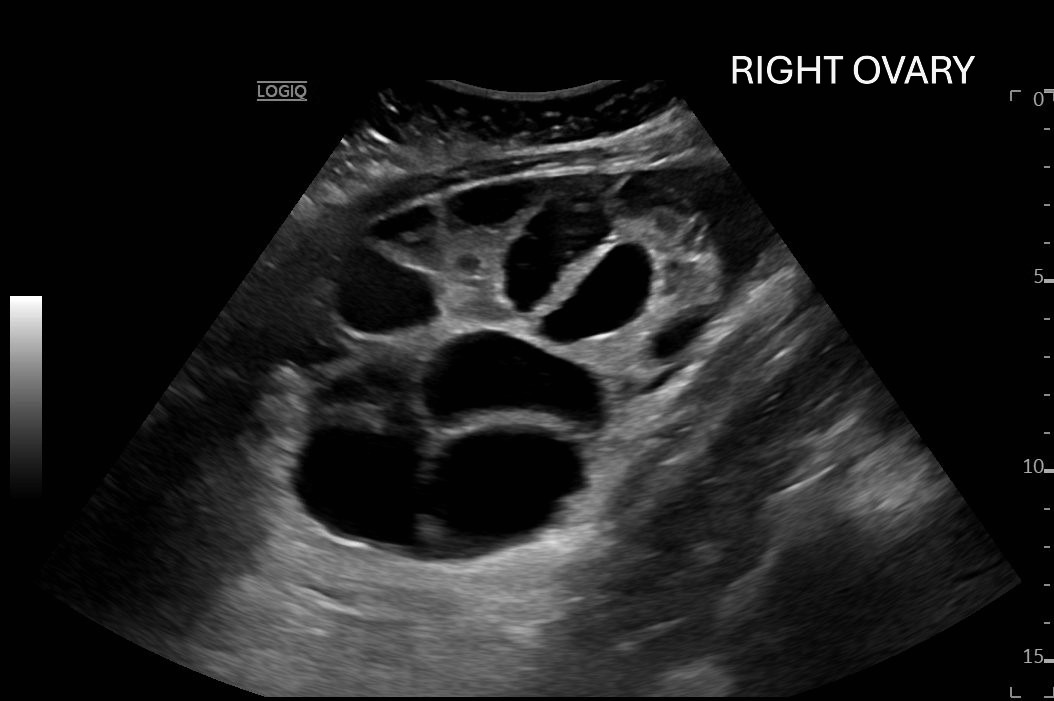

Supplement: Supplementary file 9 [file 11-2-V23-Supp9.JPG]

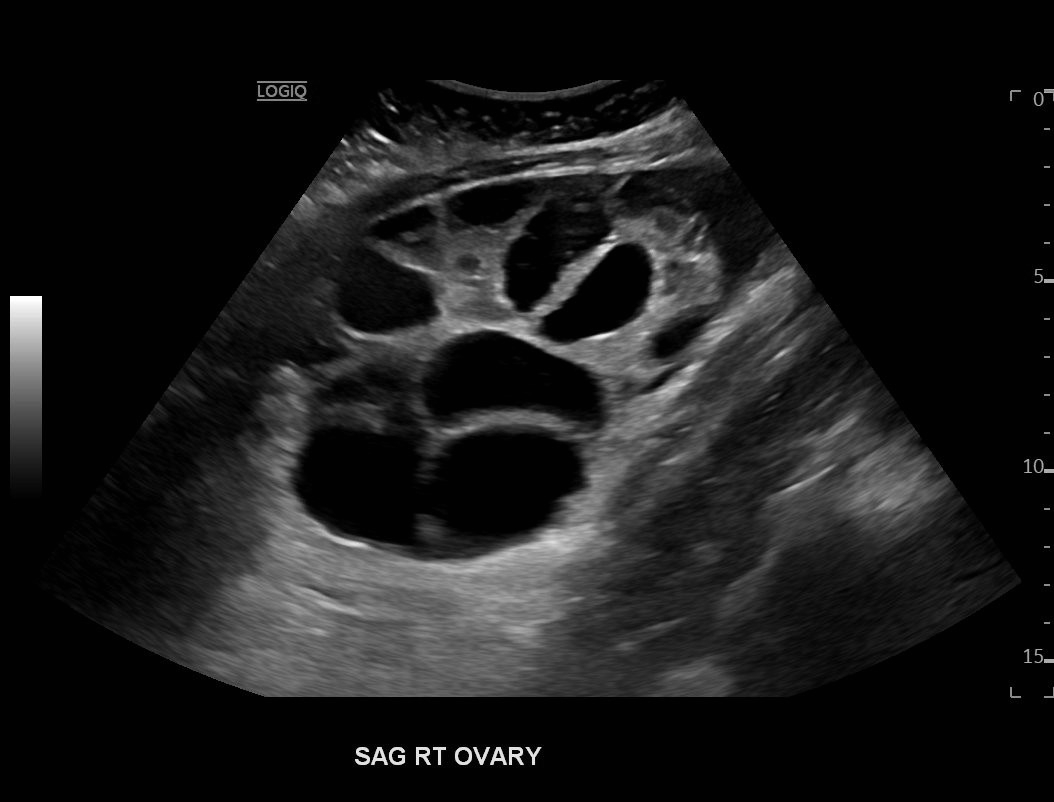

Supplement: Supplementary file 10 [file 11-2-V23-Supp10.jpg]

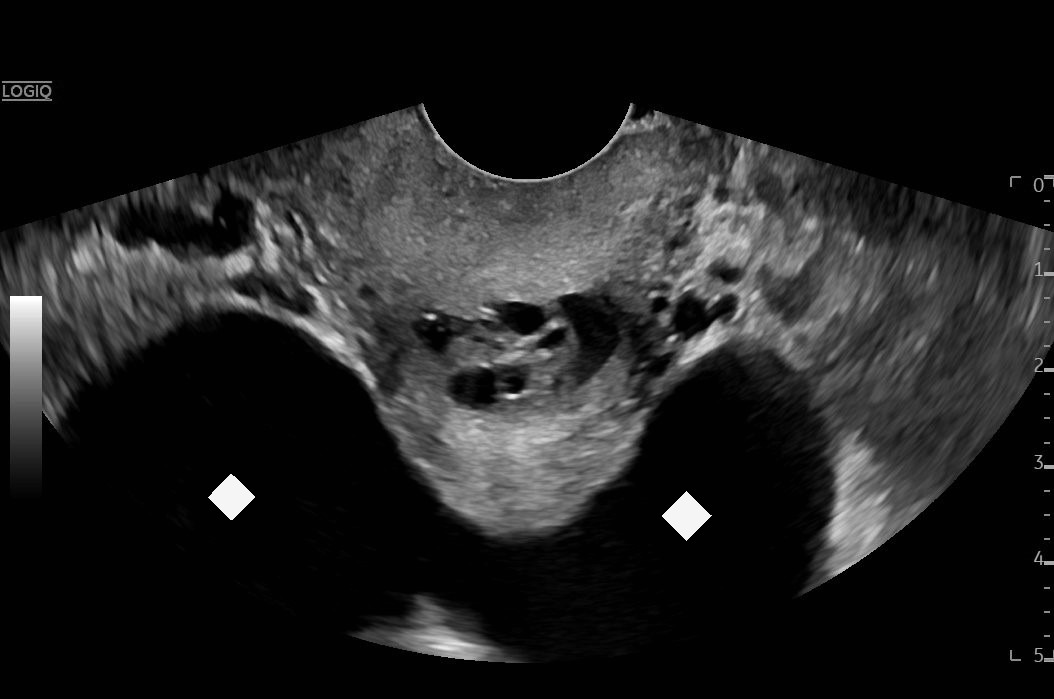

Supplement: Supplementary file 11 [file 11-2-V23-Supp11.JPG]

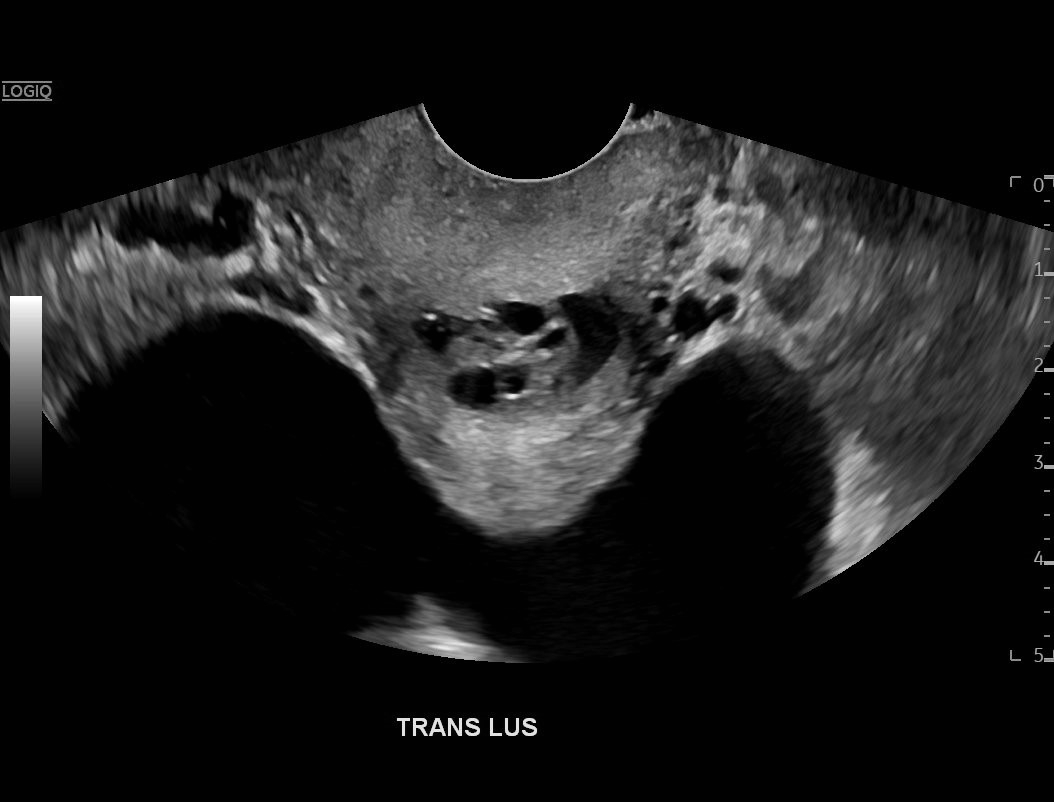

Supplement: Supplementary file 12 [file 11-2-V23-Supp12.jpg]
